# Local Ion Densities can Influence Transition Paths of Molecular Binding

**DOI:** 10.3389/fmolb.2022.858316

**Published:** 2022-04-26

**Authors:** Nicole M. Roussey, Alex Dickson

**Affiliations:** ^1^ Department of Biochemistry and Molecular Biology, Michigan State University, East Lansing, MI, United States; ^2^ Department of Computational Mathematics, Science, and Engineering, Michigan State University, East Lansing, MI, United States

**Keywords:** free energy, binding affinity, molecular dynamics, weighted ensemble, ligand unbinding, mechanisms, SAMPL system

## Abstract

Improper reaction coordinates can pose significant problems for path-based binding free energy calculations. Particularly, omission of long timescale motions can lead to over-estimation of the energetic barriers between the bound and unbound states. Many methods exist to construct the optimal reaction coordinate using a pre-defined basis set of features. Although simulations are typically conducted in explicit solvent, the solvent atoms are often excluded by these feature sets—resulting in little being known about their role in reaction coordinates, and ultimately, their role in determining (un)binding rates and free energies. In this work, analysis is done on an extensive set of host-guest unbinding trajectories, working to characterize differences between high and low probability unbinding trajectories with a focus on solvent-based features, including host-ion interactions, guest-ion interactions and location-dependent ion densities. We find that differences in ion densities as well as guest-ion interactions strongly correlate with differences in the probabilities of reactive paths that are used to determine free energies of (un)binding and play a significant role in the unbinding process.

## 1 Introduction

Atomistic simulations are a broadly used method to better understand the microscopic interactions that govern ligand binding and unbinding and to calculate critical values such as transition rates and free energies. Both rates and free energies can in principle be computed with straightforward molecular simulations, starting in either the bound or unbound state. However, the cost required to simulate binding transition paths is typically prohibitive due to high energetic barriers separating the bound and unbound states. To overcome these barriers, a variety of enhanced sampling techniques can be employed, which commonly require the use of a predefined reaction coordinate: a single collective variable that describes the progress of the (un)binding reaction.

The use of proper reaction coordinates can lead to improvements in the convergence of free energies for enhanced sampling methods Tiwary and Berne (2016) and is necessary for accurate path-based free energy calculations in biological systems Zhang and Voth (2011). Many methods have been developed to seek out optimal reaction coordinates including but not limited to VAMPnets Mardt et al. (2018), DiffNets Ward et al. (2021), Deep-TICA Bonati et al. (2021), SGOOP Tiwary and Berne (2016), and AMINO Ravindra et al. (2020). All of the above methods construct a reaction coordinate from a set of candidate features that are either predefined or require user intuition of the (un)binding process.

Significant effort has been dedicated to understanding the role of water in the ligand (un)binding process, including binding pocket solvation effects and bulk and single molecule effects Chau (2004); Tiwary et al. (2015); Maurer and Oostenbrink (2019); Rizzi et al. (2021). Water molecule density has been included in reaction coordinates through the utilization of Deep-LDA Bonati et al. (2020). This method successfully found a complex reorganization of the water structure in unbinding for use as a reaction coordinate and has been able to produce accurate binding free energies Rizzi et al. (2021). The role of ions along molecular binding pathways is much less understood. Ion distributions surrounding molecules such as double stranded DNA Kolesnikov et al. (2021) and RNA Auffinger et al. (2004) have been studied and it has been found that ion affinity for molecules such as cyclodextrins and DNA is dependent on the force field used Erdos et al. (2021) as well as the water model employed Kolesnikov et al. (2021). A difference in unbinding rates has been found between implicit and explicit ions in simulation, with implicit ion representations overestimating unbinding rates across a broad range of ion concentrations Erbas et al. (2018). However, it appears that little is known about the effects of changes in ion densities along ligand (un)binding pathways.

**FIGURE 1:**
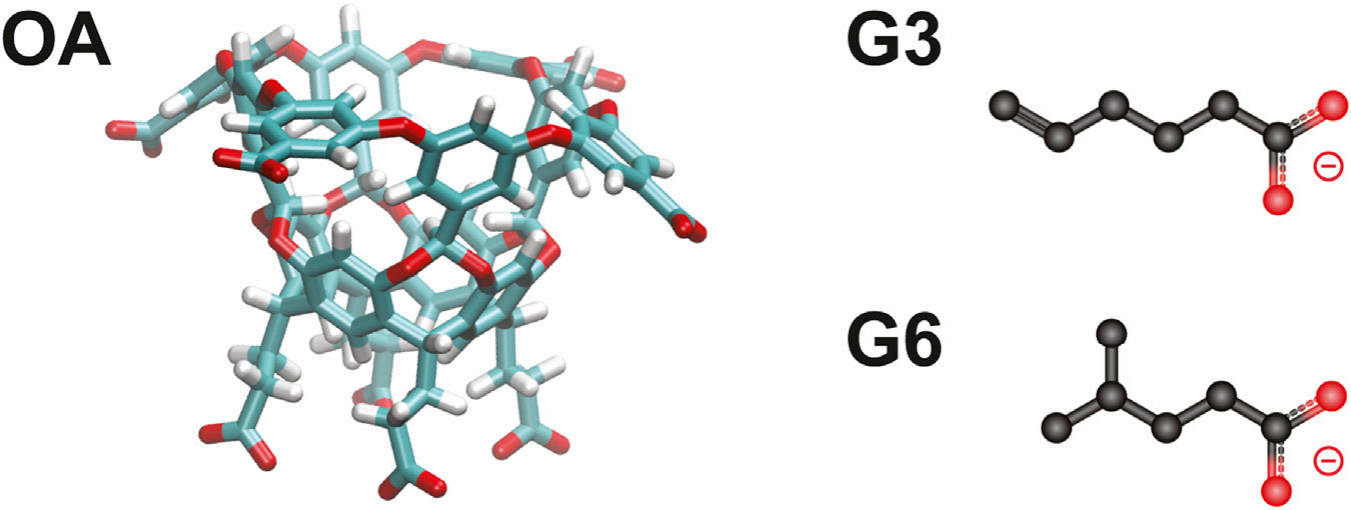
The OA-G3 and OA-G6 systems. The OA host molecule (left). The G3 (top right) and G6 (bottom right) guest molecules.

Recent studies have demontrated that adaptations of the weighted ensemble method Huber and Kim (1996); Dickson and Brooks, (2014); Donyapour et al. (2019) can efficiently generate ligand binding and unbinding pathways that can then be used to determine rates and binding free energies Dixon et al. (2018); Lotz and Dickson (2018b); Hall et al. (2020). Specifically, an extensive analysis was conducted on a series of host-guest systems containing small, organic guest molecules (“G3” and “G6”) interacting with “octa-acid” hosts (“OA”) (Figure 1), which were originally part of the SAMPL6 (Statistical Assessment of the Modeling of Proteins and Ligands) SAMPLing challenge Rizzi et al. (2018, 2020). The REVO variant of the weighted ensemble method allowed for efficient generation of large numbers of binding and unbinding events without employing biasing forces that could perturb the (un)binding mechanism. This is notable as mean first passage times of unbinding ranged up to hundreds of seconds for these systems. It accomplishes this by running an ensemble of trajectories and periodically “resampling” this ensemble to shift computational emphasis toward unique trajectories that are moving towards a target state, and adjusting the probabilities of the trajectories accordingly. As a result, each unbinding pathway has an associated statistical weight (ranging from 10^–12^ to 10^–6^) that governs how strongly it contributes to the calculation of observables, including the unbinding rate constant, *k*_*off*_.

During these resampling steps, only the geometric relationship between the host and guest molecules was used; the positions of the water molecules and ions were neglected. Here, a time- and probability-dependent analysis of solvent based features including water and ions is presented for unbinding trajectories from the OA-G3 and OA-G6 SAMPL systems. We explore the significant differences in guest-ion interactions between high- and low-probability unbinding events, also referred to as “exit points”, as well as differences in spatial arrangements of ions during unbinding. In these simulations, we have found that the generation of the most probable reactive paths requires fluctuations toward low ion densities within certain regions of the simulation box, particularly in the space immediately above the binding pocket. Differences in these ion densities along transition paths are associated with up to 10^6^-fold differences in unbinding probabilities, which motivates the future inclusion of ion densities in (un)binding progress variables.

## 2 Materials and Methods

### 2.1 Weighted Ensemble Sampling

**FIGURE 2:**
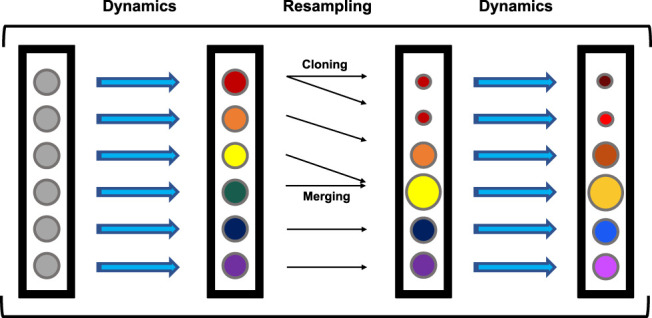
General WE Framework. Every circle represents a trajectory in the ensemble. Colors represent conformations and circle size represents probability, with all trajectories beginning with the same conformation and probability. Trajectories are run for a predetermined number of steps (dynamics), followed by a resampling step containing merging and cloning procedures. This cycle repeats until the end of the simulation.

The simulations analyzed here were previously generated Dixon et al. (2018); Hall et al. (2020) with a variant of the weighted ensemble (WE) Huber and Kim (1996) method called “REVO” Donyapour et al. (2019) utilizing the Wepy Lotz and Dickson (2020) software package. A generalized framework for WE is as follows (Figure 2). WE uses an ensemble of trajectories that are evolved forward in time in a parallel fashion. Each trajectory carries with it a statistical weight (*w*) that governs the extent to which it contributes to ensemble averages. Generally, WE simulations include two main steps: 1) An MD simulation step that moves trajectories forward in time by a predetermined time interval, and 2) a resampling step that include cloning and merging operations. Resampling is designed to both use cloning to increase the number of trajectories that have a desirable value for a feature of interest, and to decrease redundancy by merging trajectories that are similar based on the feature of interest. Together, this process aims to diversify the trajectories within the ensemble with the goal of increasing the probability of sampling rare or long-timescale events of interest for a given system. When cloning a trajectory, two new independent trajectories with the same conformation are created with half the probability, or weight (*w*) of the original. Merging two trajectories *A* and *B* leads to the creation of trajectory *C* with weight *w*_*c*_ = *w*_*a*_ + *w*_*b*_. Trajectory *C* inherits either conformation *A* or *B* with a probability proportional to *w*_*a*_ or *w*_*b*_, respectively.

A central feature of a WE simulation is the resampling function (also referred to as a “resampler”) that determines which trajectories are selected for cloning and which are selected for merging. The resampler takes in an initial set of trajectories and returns a new set, which is the outcome of a series of merging and cloning steps following the rules described above. These new trajectories thus have conformations that are a subset of the initial conformation set and the sum of trajectory weights is unchanged (typically equal to 1).

In order to determine transition rates, these WE simulations were run in a nonequilibrium ensemble, where trajectories are created in the bound state and terminated in the unbound state. The unbound state was defined using a boundary condition (BC) that is satisfied when the minimum host-guest distance is greater than 1.0 nm, following previous work Lotz and Dickson (2018a). When the BC is reached, the trajectory contributes to the reactive flux calculation according to its weight at the time of crossing, which we refer to as its “exit point probability”. The exit point probability can be anything between the minimum and maximum values set when the simulation was run. An exit point or unbinding event being considered “high-weight” or “low-weight”is relative, with this being dependent on the weights of all exit points within the dataset. The weights of trajectories vary because they are changed during the resampling steps that are done between rounds of dynamics in the weighted ensemble algorithm.

### 2.2 Resampling of Ensembles by Variation Optimization

Resampling of Ensembles by Variation Optimization (REVO) Donyapour et al. (2019) is a resampling algorithm for use with Wepy that works by maximizing a function called the **trajectory variation** (*V*). *V* is a scaled sum of the all to all pairwise distances between trajectories in the ensemble (Eq. (1)), where *d*_*ij*_ is the distance between trajectory *i* and trajectory *j* and *V*_*i*_ is the variation for trajectory *i*.

$$\begin{array}{c} V=\underset{i}{\sum }V_{i}=\underset{i}{\sum }\underset{j}{\sum }{\left(\frac{d_{ij}}{d_{⋆}}\right)}^{\alpha }\phi_{i}\phi_{j} \end{array}$$ (1)

The measurement of distance between two trajectoies can be arbitrarily defined in the REVO method. In this case it was defined as the root mean squared deviation of the ligand after aligning the host molecules. As the host molecules have four-fold symmetry, four separate distances were calculated after aligning the hosts in the four symmetrically-equivalent postitions, upon which the smallest such distance was used for *d*_*ij*_. *ϕ*_*i*_ is a non-negative function referred to as a “novelty” that signifies the importance of individual trajectories. In this work is was solely a function of walker weight. *d*_⋆_, the “characteristic distance” is the average distance after one cycle of dynamics, and is only used to make the varation function unitless. The *α* parameter balances the value of the distance and novelty terms and was set equal to 4. Other methodological details pertinent to data generation are available in Ref. Dixon et al. (2018) and Ref. Hall et al. (2020). The overall goal of REVO is to optimize the value of *V* by cloning trajectories with a high value of *V*_*i*_ and merging trajectories with a low value of *V*_*i*_. See Ref. Donyapour et al. (2019) for more details of the REVO method.

### 2.3 Dataset Information

The weighted ensemble data used for this analysis comes from papers published in 2020 Hall et al. (2020) (the primary OA-G6 data set) and 2018 Dixon et al. (2018) (OA-G3 data set and a secondary OA-G6 data set). Briefly, the primary OA-G6 data set contains 10 simulations with 48 trajectories each and 1,500 cycles per trajectory that begin in the initial OA-G6-0 pose provided in the SAMPL6 SAMPLing challenge Rizzi et al. (2020). The 2018 data sets contain five simulations each with 48 trajectories and 2000 cycles per trajectory, each beginning at one of the five initial poses for the corresponding system. Reactive paths begin in the bound state and end in the unbound state when a BC is hit. The BC is defined as a 1.0 nm minimum distance between the host and guest molecules.

## 3 Results

**FIGURE 3:**
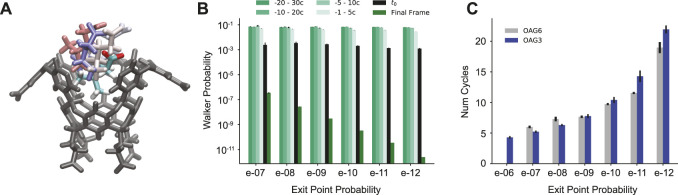
Analysis of *t*_0_ poses. **(A)** The OA host molecule with the G6 ligand in the starting pose (multi-color) and example *t*_0_ poses (pastels). Some atoms from the host have been removed in A for clarity. **(B)** Average probabilities from -30 cycles to the final unbinding event organized by unbinding probability for the 2020 OA-G6 data set. **(C)** The average number of cycles between *t*_0_ and the unbinding event for OA-G3 (blue) and the 2020 OA-G6 data set (gray) organized by unbinding probability.

We find that each reactive path can be split into two phases: 1) initial departure from the bound state, and 2) full separation of the host and guest. There are often many cycles between the guest physically leaving the binding pocket of the host and the BC being hit. It was determined that in all of the reactive paths generated, a center-of-mass (COM) to COM distance of 0.7 nm indicated an irreversible transition between these two parts (Supplementary Figure S1). This can be seen as a physical “commitment to unbinding” point after which rebinding does not occur, where the guest has just been released from the partially solvated binding pocket (Figure 3A). The cycle corresponding to this point is found for all reactive paths and used for analysis; we refer to this point as *t*_0_.

When the BC is hit for the reactive paths, the unbinding probabilities varied between 10^–12^ and 10^–6^ for OA-G3 and between 10^–12^ and 10^–7^ for OA-G6. The low probability exit points are highly abundant for both OA-G6 and OA-G3, whereas the high probability exit points occur with a very low frequency for both systems. Overall, the number of exit points *increases* as the probability of the exit points *decreases* (Table 1).

**TABLE 1:** The number of observed unbinding events grouped by exit point probability. The OA-G6 row corresponds to the OA-G6 2020 dataset.

	10^–6^	10^–7^	10^–8^	10^–9^	10^–10^	10^–11^	10^–12^
OA-G6	0	7	17	112	359	652	2220
OA-G3	10	18	88	195	483	1,116	4103

At and before the *t*_0_ point, the probabilities of the reactive paths are roughly the same, with a value of 10^–3^ with only the probabilities following *t*_0_ varying based on exit point probability (Figure 3B). The number of cycles between *t*_0_ and the unbinding event also correlates to the exit point probability, with high probability exit points having ∼5 cycles between the two points, and ∼20 cycles for low probability exit points (Figure 3C). There is a steady increase in the average number of cycles between *t*_0_ and the unbinding event as the probability of the trajectories decreases.

**FIGURE 4:**
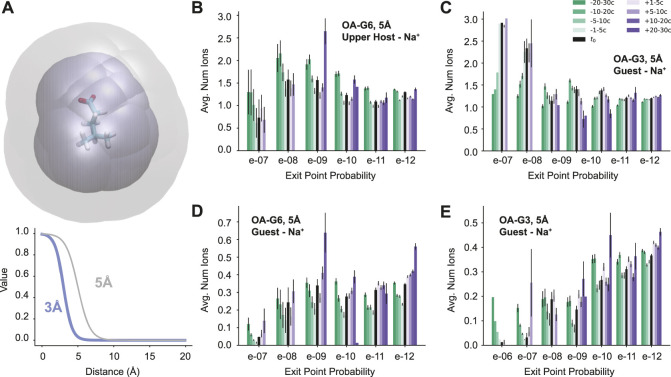
Feature Analysis. **(A)** A visualization of the region of space considered for the guest-ion features using the G6 ligand. The maximum distance for the 5 Å scale is in gray and the 3 Å scale is in blue (top). The two logistic functions used to determine the molecule counts (bottom). **(B–E)** Molecule counts for Na^+^ ions with results organized by both time and exit point probability. The legend in C applies to all four plots. The average total ion count (5 Å scale) around the upper negative charges of the host for **(B)** OA-G6 and **(C)** OA-G3. The average total ion count (5 Å scale) around the guest for **(D)** OA-G6 and **(E)** OA-G3. OA-G6 results correspond to the 2020 data set.

These differences prompt the question: are there differences in physical features associated with this large variation in exit point probability? To answer this question, a set of physical features was chosen and the values of those features were calculated for every cycle of every reactive path generated. The features in question include the number of waters in the binding site of the host, the number of ions around the upper negative charges of the host molecule, the number of ions around the guest, and the number of waters around the guest molecule (Figure 4A). To calculate these features, a continuous logistic function was used: $f\left(r\right)=1-\frac{1}{1+\left(e^{-S\left(r-r_{0}\right)}\right)}$ , where *r* is the minimum atomic distance between the two entities. We use two different sets of values for the interaction radius (*r*_0_) and steepness (*S*) parameters: *r*_0_ = 3 Å, *S* = 17 or *r*_0_ = 5 Å and *S* = 12) (Figure 4A). The sum of *f*(*r*) across all ions (or waters) is a continuous count of the number of molecules of that species surrounding the host (or guest) for that cycle.

It was found that some features were consistent or had only a slight variation across all exit point probabilities, such as the number of binding site waters and the number of waters surrounding the guest molecule (Supplementary Figure S2). However, some features were found to show trends that differentiated the high- and low-probability exit points. These features included the total number of positive ions surrounding the upper negative charges of the host (Figure 4B,C) and the number of positive ions surrounding the guest molecule (Figure 4D,E). In both OA-G6 and OA-G3 there is a general trend of the number of ions surrounding the upper negative charges of the host increasing as the exit point probability *increases*, although this is not observed for 1*e* − 7 exit points in the OA-G6 dataset. There is also a clear trend of increasing guest-Na^+^ interaction as the exit point probability *decreases* including before, at, and after the *t*_0_ point. Similar trends were observed for features on the 3 Å scale (Supplementary Figure S3).

**FIGURE 5:**
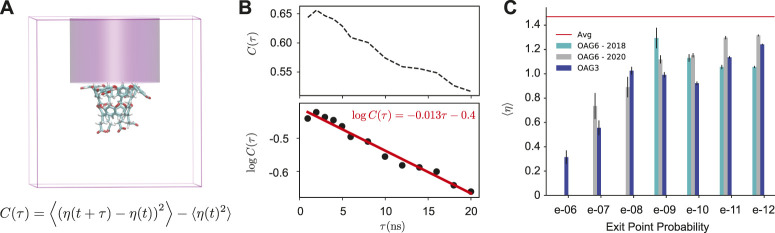
Ion Density Analysis. **(A)** A diagram showing the simulation box and the cylindrical space above the host where the number of ions (*η*(*t*)) is determined. The equation for calculating the autocorrelation of this quantity (*C*(*τ*)) is shown. **(B)** An autocorrelation plot of the cylindrical ion density (*η*(*t*)) is calculated using all reactive and non-reactive trajectory data. **(C)** The average number of ions in the cylinder space above the host for OA-G3 (dark blue), OA-G6 (2020, gray), and OA-G6 (2018, cyan). The average from 4500 random simulation cycles is shown in red.

As we find that the interaction between the guest and Na^+^ ions correlates with the probability of the unbinding trajectories, we now examine Na^+^ ion densities in the region of space directly above the host. Specifically, we examine a cylindrical region of space beginning immediately above the host and ending at the top of the box (Figure 5A). We find that this region is critical to determine the outcome of dissociation trajectories that have reached *t*_0_. The autocorrelation of ion density in this region (*C*(*τ*)) is surprisingly long-lived; it follows a single exponential decay with a timescale of 77 ns (Figure 5B).

Figure 5C shows the average number of ions in the cylinder for cycles [*t*_0_ − 3, *t*_0_ + 3] for each reactive trajectory. An average of 1.47 ions was found in upper cylinder space when averaged over all available data (including reactive and non-reactive trajectories). The cylinder ion densities of *reactive trajectories* were found to be significantly lower than the bulk average regardless of exit point probability. A striking relationship was observed between this ion density and the exit point probability that was consistent across all data sets with highly weighted exit point probabilities (Figure 5C), where highly-weighted exit points had a significantly lower average number of ions in the cylinder. Overall, highly weighted exit points had less ions above the host, and subsequently near the guest at *t*_0_, with this number gradually increasing as the exit point probability decreased (Supplementary Figure S4).

To explain these findings, we first analyzed the electrostatic forces on the guest molecule for all OA-G6 reactive trajectories at the *t*_0_ point for one ensemble of the OA-G6 2020 data set. This was done by first removing all forces from the system other than the nonbonded (electrostatic) forces. Then the force on the ligand was determined at key points along the unbinding trajectories and average forces were determined for each exit point probability group. Results are shown for the initial bound cycles (cycles 0–6) and for the *t*_0_-surrounding cycles used for the cylinder-ion analysis (Supplementary Figure S5). We find that the net electrostatic force is pushing the guest outward from the host, and that the magnitude of this force is about 20 kJ/mol/Å higher in the initial pose (80 kJ/mol/Å) than it is at *t*_0_ (60 kJ/mol/Å). No significant difference is found for exit points of different weights for both the overall magnitude of the electrostatic force or the *z*-axis contribution to the force (Supplementary Figure S5A,B). We found no significant difference at *t*_0_ across all exit point probabilities despite the difference in cylinder ion-occupancy.

**FIGURE 6:**
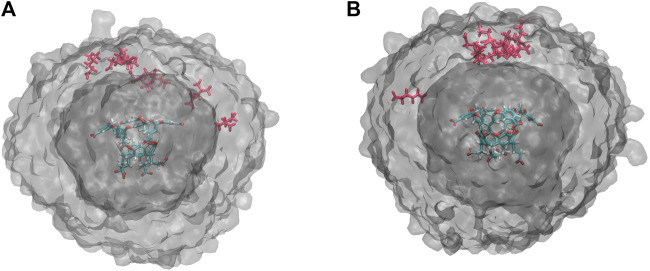
Exit Point Analysis. **(A)** Unbinding event locations for exit points with probabilities 10^–7^ (red) and 10^–12^ (gray VolMap) for OA-G6. **(B)** Unbinding event locations for exit points with probabilities 10^–6^ (red) and 10^–12^ (gray VolMap) for OA-G3. The surfaces show a density contour (Isoval) of 0.0001 in both panels.

An alternative explanation is that differences in occupancy change the likelihood of ion interaction after the *t*_0_ point. This is consistent with our observations in Figure 4D,E and would increase the number of cycles required to hit the BC (Figure 3C) as well as the extent of their exploration of the simulation box. Exit point locations were determined for both the highest and lowest probability exit points for both OA-G6 (10^–7^ and 10^–12^) and OA-G3 (10^–6^ and 10^–12^). For both systems, it was found that for high probability exit points, most guest molecules reach the BC directly above the host, whereas the low probability exit points hit the BC at a wide distribution of points surrounding the host molecule (Figure 6).

## 4 Discussion

In summary, we find that location-dependent ion densities play a significant role in the unbinding process for the OA-G6 and OA-G3 systems. These systems are widely used for both the testing and development of force fields and numerous computational methods Rizzi et al. (2020, 2018); Dixon et al. (2018); Papadourakis et al. (2018); Song et al. (2018); Yin et al. (2016) necessitating a thorough understanding of the mechanics of their unbinding. It is likely that ion densities play such a prominent role due to the charged nature of these systems (−8 for the host and −1 for the guest). Similar effects might also be observed in biological systems with even more significant charge densities such as calsequestrin Yano et al. (2009), a protein necessary for muscle relaxation/contraction, with a net charge of -64, as well as systems with nucleic acids, which have a charge of −1 per nucleotide.

Further exploration and utilization of the effects of ion densities on ligand (un)binding could be done via various methods. Constraints on spatial densities of ions could be included in simulations to further examine the relationship between ion densities and unbinding rates or free energies. One possible strategy would be to conduct 2D Umbrella Sampling Park and Im (2013); Dickson et al. (2015) simulations that include a direct descriptor of (un)binding, such as the host-guest center-of-mass distance, and the ion density added as a second collective variable. Ion densities (and other features of interest) could also be utilized for resampling purposes for weighted ensemble simulations for the determination of distances between trajectories. This could encourage cloning operations of trajectories with ions in desirable locations, potentially allowing for more efficient generation of high probability unbinding events.

In weighted ensemble sampling, the equilibrium probability of a state is obtained by summing over the weights of all trajectories that have visited that state. This is similarly true for reactive paths: the overall probability of a path is determined by a weighted sum of trajectories. The analysis above breaks down a reactive trajectory set by weight, but it is important to note that relationship between the weight of a trajectory and the probability of the corresponding reaction path is not one-to-one. While high-weight trajectories in general sample from high-probability regions of space, it is possible that a low-weight trajectory could visit a high-probability reaction path. For this reason, we should consider the low-probability trajectories (e.g. *p* = 10^–12^) as a heterogeneous group that could contain observations of high-probability reaction paths. However, the high-weight trajectories (by definition) correspond only to high-probability paths.

Overall, these results suggest that greater attention may be required for ligand-ion interactions across various simulation methods, including those that require a predefined reaction coordinate. We find that there are many microscopic trajectories that contribute to the unbinding path ensemble, some of which are much more likely than others. Methods that only sample unlikely reactive paths could have difficulty computing accurate measurements of transition rates and free energies. In addition, incorrect transition states (including inaccuracies in solvent degrees of freedom) can lead to incorrect hypotheses about the molecular interactions that govern kinetics. This work underscores the importance of proper consideration of ion densities along unbinding pathways, especially for charged systems.

## Data Availability

The data analyzed in this study is subject to the following licenses/restrictions: The datasets analyzed in this study are available on request to the corresponding author. Requests to access these datasets should be directed to alexrd@msu.edu.
