# Supplementary material for: Local Ion Densities can Influence Transition Paths of Molecular Binding
Source: Front Mol Biosci. 2022 Apr 26;9:858316. doi: 10.3389/fmolb.2022.858316 (PMC9086317; doi:10.3389/fmolb.2022.858316)
Supplement: Supplementary file 1 [file DataSheet1.PDF]

Supplementary Material

1 SUPPLEMENTARY DATA

1.1 Determination of t_0

The center-of-mass to center-of-mass (COM-to-COM) distance between the host and guest molecule was plotted for several hundred unbinding events (Fig. S1). These unbinding events cover the full range of unbinding probabilities. An example of a rebinding event is seen in pink at ~ 300 cycles. Rebinding events for reactive trajectories do not occur once a COM-to-COM distance of 0.7nm is reached.

1.2 Other solvent-based features

Other features were analyzed for these systems including the total number of waters in the binding site. This included a hard cutoff at 0.64nm from the center of the binding pocket to prevent counting waters outside of the binding pocket (Fig. S2A and B). There is a slight dependence on exit point probability for the number of waters in the binding site for both OA-G6 and OA-G3. The total number of waters around the guest molecule was also determined (Fig. S2C and D).

Analysis was also done using the 3Å based logistic function for the upper host negative charges and Na^+ interactions (Fig. S3A and B) as well as the guest and Na^+ interactions (Fig. S3C and D) for both OA-G6 and OA-G3. No significant differences were found for this analysis other than decreased molecule counts, which is to be expected.

1.3 Ion Density By Z-Axis

Here, we examine the average number of ions above the host, at host-level, and below the host at the t_0 adjacent cycles ($[t_0 - 3, t_0 + 3]$), organized by exit point probability (Fig.S4).

1.4 Electrostatic force analysis of unbinding paths with different weights

Here, we examine the electrostatic forces on the guest molecule for the reactive trajectories in one ensemble of the OA-G6 2020 data set (Fig. S5) as previously described in the main text.

2 SUPPLEMENTARY FIGURES

2.1 Figures

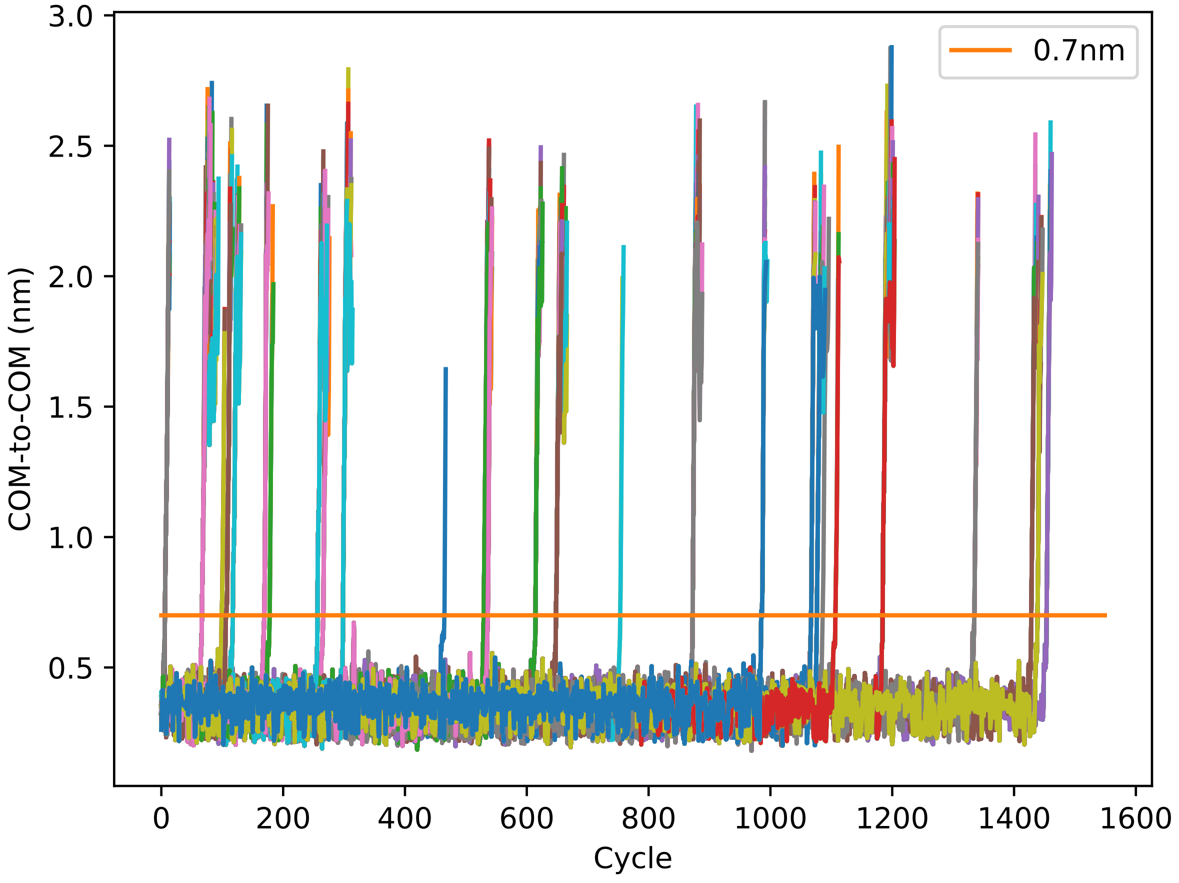

Figure S1. Center of Mass to Center of Mass Distances. The COM-to-COM distance between the host and guest is shown for several hundred unbinding events for OA-G6. Rebinding only occures below 0.7nm (orange line).

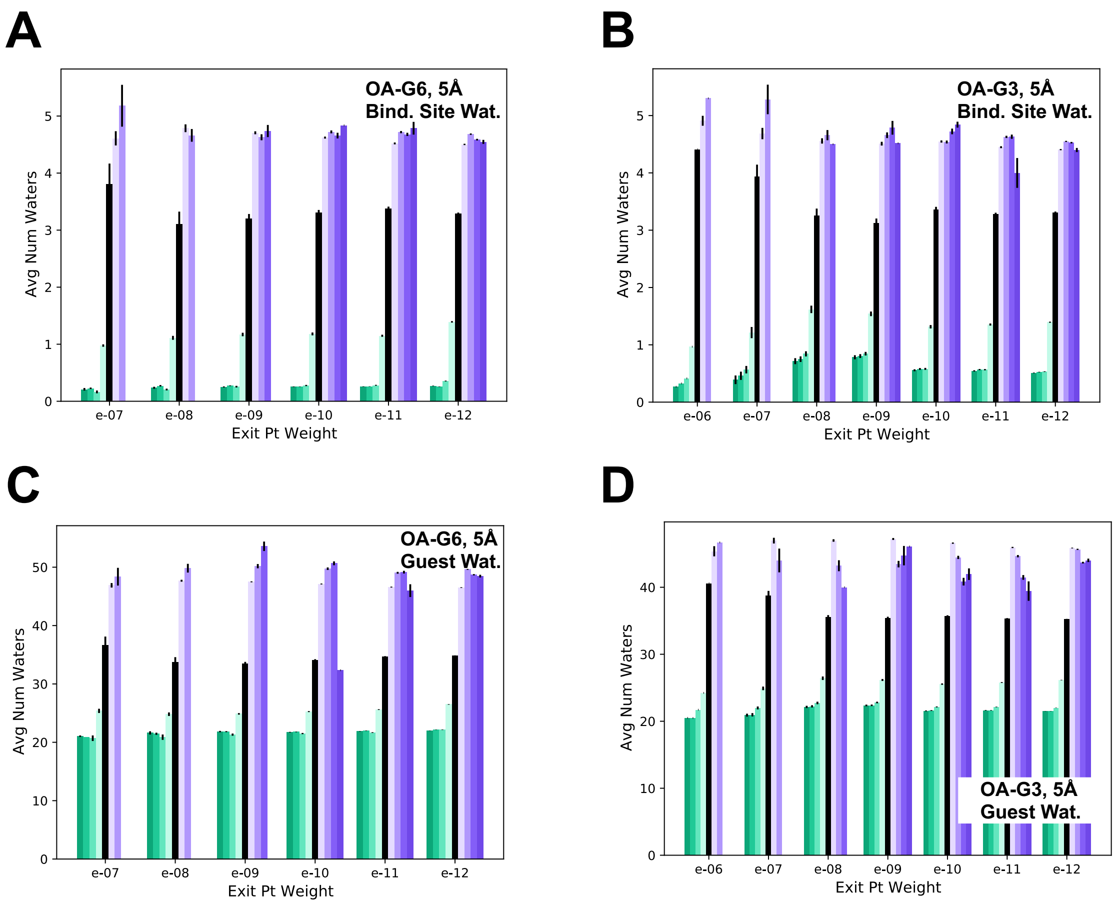

Figure S2. 5Å **Analysis of Water Based Features**. Molecule counts for waters with results organized by exit point probability. The legend in Fig. S3 applies to all four plots. The average total water count (5Å) in the binding site of the host for A) OA-G6 and B) OA-G3. The average total water count (5Å) around the guest for C) OA-G6 and D) OA-G3.

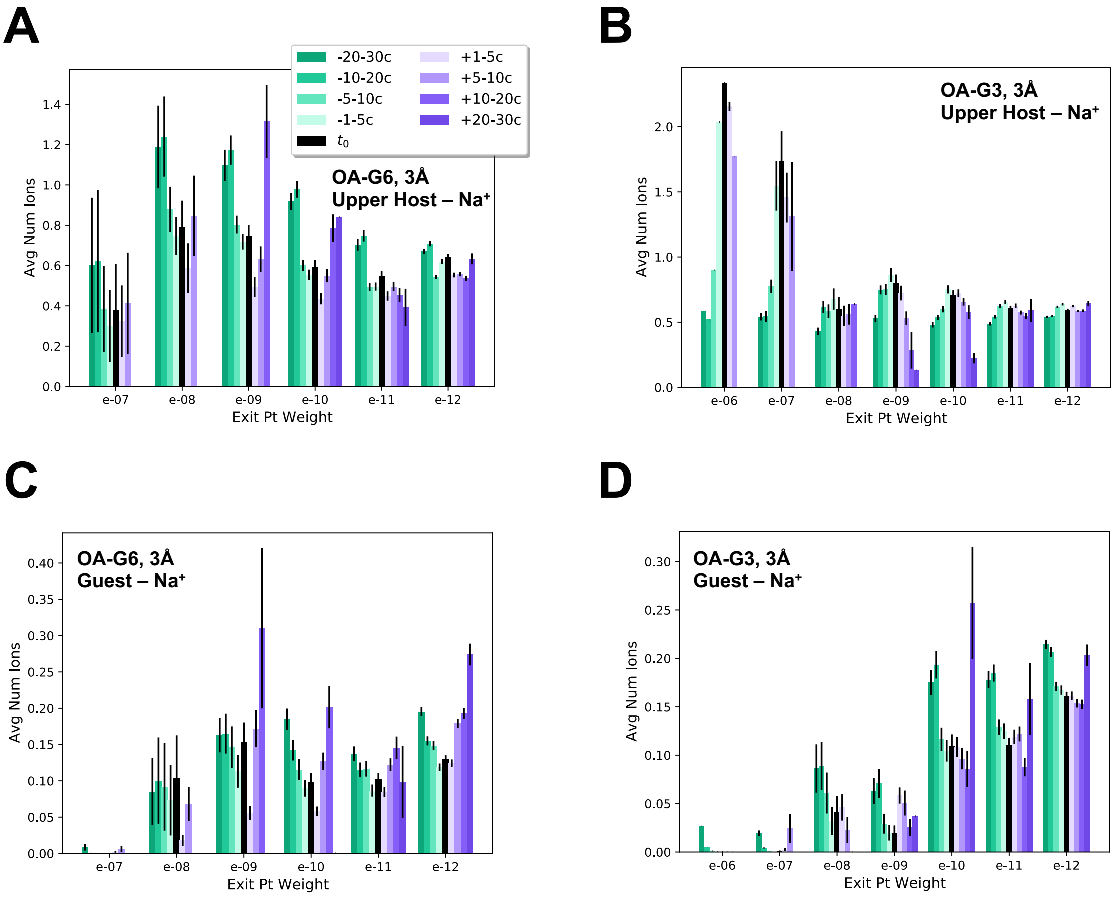

Figure S3. 3Å **Analysis of Ion Features**. Molecule counts for Na^+ ions with results organized by exit point probability. The legend in A applies to all four plots. The average total ion count (3Å) around the upper negative charges of the host for A) OA-G6 and B) OA-G3. The average total ion count (3Å) around the guest for C) OA-G6 and D) OA-G3.

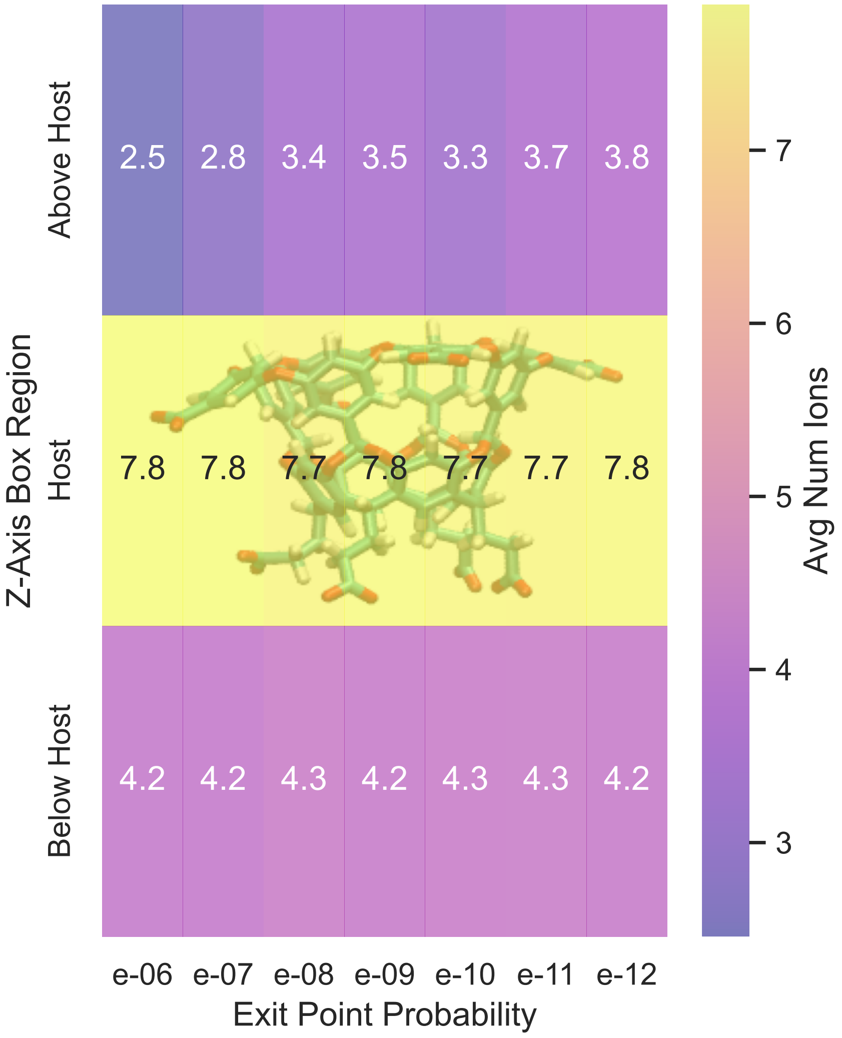

Figure S4. Z-Axis Ion Density. The average number of ions in the region of space above the host, at host level, and below the host for the t_0 adjacent cycles ($[t_0 - 3, t_0 + 3]$) for all OA-G3 exit points. Corresponding errors by weight are as follows: $+/-$ $0.78401176, 0.77167242, 0.52423166, 0.35946138, 0.21856345, 0.14066211, 0.07147705$.

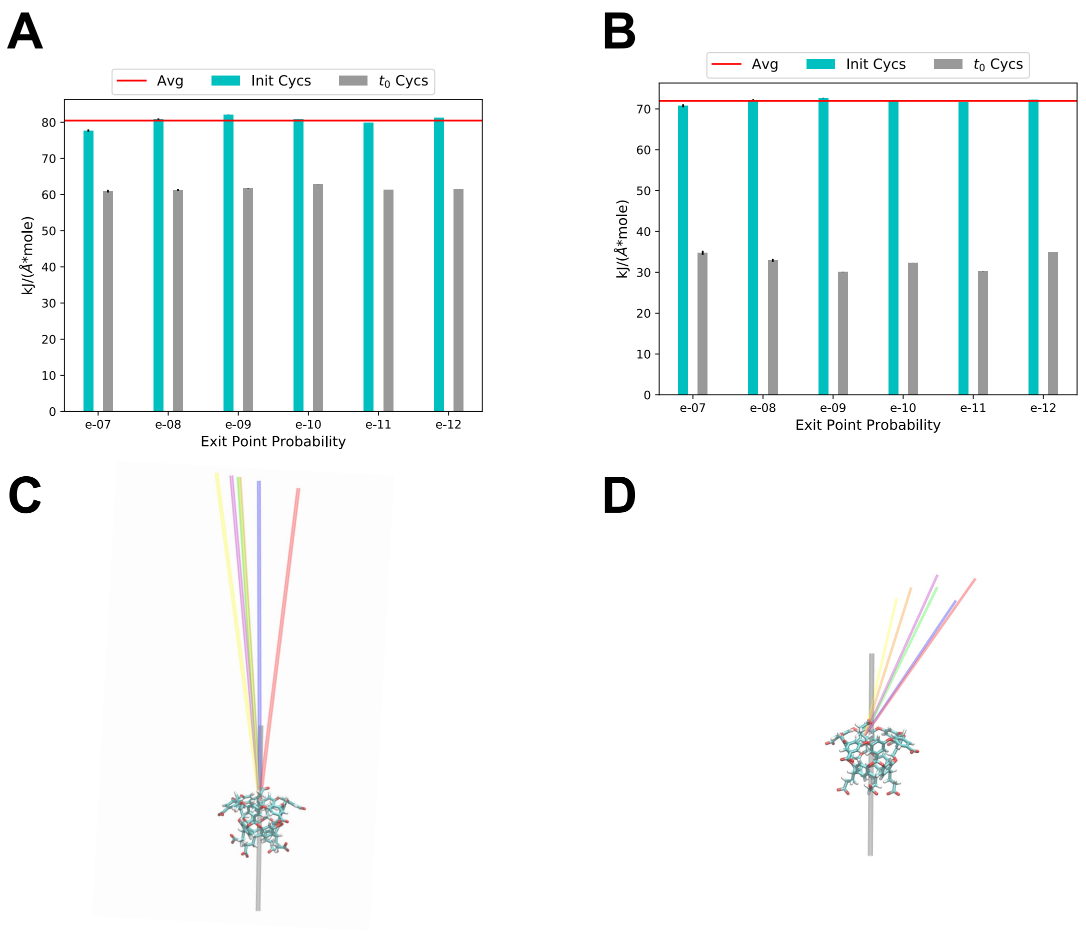

Figure S5. Electrostatic Analysis. A) The magnitude and B) the z-axis contribution of the electrostatic force for the initial cycles (cyan) and t_0 cycles. The average of the initial cycles is shown as a horizontal red line. C) The force vectors for the initial cycles. D) The force vectors at t_0. For both C and D, the gray bar is the Z axis. Red, orange, yellow, green, blue and violet correspond to 10^{-7} to 10^{-12} exit point probabilities.
